# COVID-19 and gender-based violence service provision in the United States

**DOI:** 10.1371/journal.pone.0263970

**Published:** 2022-02-16

**Authors:** Rachel Sapire, Jennifer Ostrowski, Malia Maier, Goleen Samari, Clarisa Bencomo, Terry McGovern

**Affiliations:** 1 Heilbrunn Department of Population and Family Health, Columbia University Mailman School of Public Health, New York, New York, United States of America; 2 Program on Global Health Justice and Governance, Columbia University Mailman School of Public Health, New York, New York, United States of America; De Montfort University, UNITED KINGDOM

## Abstract

**Introduction:**

Gender-based violence (GBV) policies and services in the United States (U.S.) have historically been underfunded and siloed from other health services. Soon after the onset of the COVID-19 pandemic, reports emerged noting increases in GBV and disruption of health services but few studies have empirically investigated these impacts. This study examines how the existing GBV funding and policy landscape, COVID-19, and resulting state policies in the first six months of the pandemic affect GBV health service provision in the U.S.

**Methods:**

This is a mixed method study consisting of 1) an analysis of state-by-state emergency response policies review; 2) a quantitative analysis of a survey of U.S.-based GBV service providers (N = 77); and 3) a qualitative analysis of in-depth interviews with U.S.-based GBV service providers (N = 11). Respondents spanned a range of organization types, populations served, and states.

**Results:**

Twenty-one states enacted protections for GBV survivors and five states included explicit exemptions from non-essential business closures for GBV service providers. Through the surveys and interviews, GBV service providers note three major themes on COVID-19’s impact on GBV services: reductions in GBV service provision and quality and increased workload, shifts in service utilization, and funding impacts. Findings also indicate GBV inequities were exacerbated for historically underserved groups.

**Discussion:**

The noted disruptions on GBV services from the COVID-19 pandemic overlaid long-term policy and funding limitations that left service providers unprepared for the challenges posed by the pandemic. Future policies, in emergency and non-emergency contexts, should recognize GBV as essential care and ensure comprehensive services for clients, particularly members of historically underserved groups.

## Introduction

Gender-based violence (GBV), an umbrella term for any harmful act that targets a person based on their socially ascribed gender, continues to be a pervasive problem for people around the world [1]. GBV persists in many forms, including sexual, physical and/or emotional violence. Globally, one in three women has experienced some form of GBV in their lifetime, with this rate likely an underestimate of the actual burden, given the sensitive nature and common under-reporting associated with GBV [2].

GBV policies and services in the United States (U.S.) have historically been underfunded and siloed from the rest of the healthcare and sexual and reproductive health service delivery system [3, 4]. Estimates suggest that the U.S spends $3.5 trillion annually on costs associated with violence against women, including medical expenses and lost wages [5]. Even before the coronavirus disease 2019 (COVID-19) pandemic, federal funding to address GBV was disproportionately low relative to GBV’s health and economic burden. GBV receives just 0.09% in total funding of the estimated annual economic burden it generates for rape and attempted rape alone, compared to 0.37% for cardiovascular disease and 2.38% for cancer, with the majority of funding directed to justice mechanisms, including criminal and legal services, rather than health services [3]. Pre-existing policy and funding constraints related to GBV health services in the U.S. provide context for how policy makers and health service providers responded to the COVID-19 crisis and, ultimately, how care was affected.

### Federal laws, regulations, and funding shaping GBV prevention and response

Federal resources for GBV are primarily provisioned through the Violence Against Women Act (VAWA) of 1994, the Family Violence Prevention and Services Act (FVPSA) of 1984, and coverage permitted by public health insurance. Together, these funding streams help shape the U.S. GBV service provision landscape.

The Violence Against Women Act (VAWA) was the first comprehensive federal piece of legislation targeting GBV and was the direct result of women’s groups’ hard-fought advocacy to secure recognition and resources for the breadth of GBV’s health and economic impacts and the lack of state-level protections to address it [6]. VAWA changed the trajectory of GBV funding and how the U.S. justice system viewed and prosecuted its crimes. Prior to its passage, domestic violence (DV) was commonly seen as a private matter, and police officers were discouraged from intervening in crimes [6]. VAWA funds are administered by the Office on Violence Against Women (OVW) under the Department of Justice (DOJ). Since 1995, OVW has awarded over $8 billion in grants to state, tribal, and local governments and non-profit organizations [7].

While VAWA is the primary federal funding mechanism for GBV criminal justice programs, the Family Violence Prevention and Services Act (FVPSA) is the primary federal funding source for GBV support services. FVPSA services support survivors of DV and their children through (1) a national DV hotline (receiving $3.5M per year for FY2011-2015), (2) the Domestic Violence Prevention Enhancement and Leadership Through Allies (DELTA) aimed at preventing DV in select communities (receiving $6M per year for FY2011-2015), and (3) direct services for victims and their families through grants to states, territories, and tribes distributed through local DV service organizations (receiving $175M per year for FY2011-2015). FVPSA funding is delivered through the U.S. Department of Health and Human Services (HHS) [8].

Total annual federal funding for GBV provisioned through VAWA and FVSPA amounted to $739 million in 2019 [7, 8]. Private sources, including foundations, are another important funding stream for GBV-related health services. Although data on foundation funding for GBV in the U.S. is limited, a Ms. Foundation survey found that funding for GBV organizations and programs from foundation grants $10,000 and above grew from 417 grants totaling $23,869,610 in 1994 (adjusted for inflation) to 1,451 grants totaling $80,333,827 in 2008 (about 10% of total annual public funding) [7–9]. This nearly 3.5 times increase is significant, but as a percentage of total foundation funding, GBV grant values only increased from 1.5% in 1994 to 1.8% in 2008 [9]. Private donors may also devote resources to GBV for only a limited period of time, destabilizing long-term service delivery [9].

Prior to the onset of the COVID-19 crisis, GBV service providers reported a need for additional resources to address the impact of GBV [9]. With funding largely from federal sources, programs are vulnerable to budget cuts during economic downturns and political shifts and are molded by federal priorities that emphasize criminalization rather than social services and advocacy efforts [3]. Consequences include a slower rate of DV crime reduction than the drop in the overall crime rate; a siloed focus on physical violence over other forms of GBV, like emotional abuse and reproductive abuse; and a contribution to the U.S.’s mass incarceration crisis, affecting mostly historically marginalized communities [4]. The most recent reauthorization of VAWA passed in the House but stalled in the Senate in 2019 and remains expired as of April 2021 due to bipartisan disagreements on new provisions of the Act. Congress continued to appropriate funds for VAWA in 2020 for the interim period, but extended stagnation may risk the services it funds in the future [6, 10].

Prior to 2010, U.S. insurance companies could categorize a history of interpersonal violence (IPV) as a pre-existing condition in order to charge a higher premium or deny coverage [11]. The 2010 Affordable Care Act (ACA) set new rules that require most insurers to cover screening and counseling for GBV at no cost and, critically, prohibited insurers from classifying GBV as a pre-existing condition to charge higher premiums or deny or revoke coverage [11]. These coverage changes have been found to increase women’s willingness to seek care and improve health service utilization for violence [11]. In addition to financial barriers to health service utilization, the literature indicates there are additional barriers for historically underserved populations. For example, LGBTQ (lesbian, gay, bisexual, transgender, and genderqueer) individuals experience stigma that may prevent them from seeking and receiving appropriate health services and immigrant communities experience challenges both due to services often offered only in English and fears of deportation upon service utilization [12–15].

Health services and access to GBV service provision are a critical element in determining survivors’ health outcomes. Exposure to violence is a significant contributor to and risk factor for chronic diseases, poor mental health, gastrointestinal disorders, and autoimmune diseases, among many other negative health outcomes, thus intervening before these health outcomes crystallize is key [3]. Evidence indicates that survivors will utilize services when confidential, accessible health services are provided, and those who utilize services are more likely to disclose violence and have greater health outcomes [16–18].

### GBV health services & COVID-19

Against this backdrop of fragmented policy and underfunded GBV service provision, particularly for historically underserved populations, in January of 2020, the first COVID-19 case in the U.S. was confirmed, with stay-at-home orders beginning in March 2020 [19]. Soon after, media sources, advocacy groups, police stations, and hotlines reported increases in GBV and at times decreased health service access as a result of COVID-19-related protective policies globally [20–23]. Academic reviews and commentaries have reported increases in violence in regions where strict social distancing measures were adopted [22–24]. Measures to combat COVID-19 have been found to increase the frequency and severity of GBV in many ways, including exacerbating risk factors for GBV, for example, mental and/or financial stress, and reducing access to support services through limited mobility, restricted access to social support mechanisms, and barriers to health services [20, 25, 26]. The majority of the existing work includes academic commentaries, and there are limited research studies on COVID-19 and GBV [21, 27–29]. Additionally, many of the research studies are from low- and middle-income countries [24, 30, 31], with some sparse information on GBV service delivery during COVID-19 in Australia [26, 32]. As such, the effects of COVID-19 on GBV service provision require urgent investigation.

This study aims to address the knowledge gap on the impact of COVID-19 policy responses on GBV service providers in the United States. In the absence of a coordinated federal response, state COVID-19 public health guidance and policy implementation largely determined on-the-ground realities for GBV service providers. Against a backdrop of existing funding stressors and concurrent sociopolitical events, we consider how state emergency policies and other COVID-19 related disruptions affect GBV service utilization. Through a mixed method approach, we analyze how COVID-19 and resulting state policies in the first six months of the pandemic affected GBV service provision.

## Materials & methods

This mixed method study consists of 1) a state-by-state emergency response policy review, 2) a quantitative analysis of a survey of U.S.-based GBV service providers, and 3) a qualitative analysis of in-depth interviews with U.S.-based GBV service providers. GBV service providers surveyed and interviewed spanned a range of organization types, populations served, and states to examine COVID-19 and related policy changes’ impact on service provision.

The state-by-state emergency policy review focused on mapping state-level actions related to nonessential service closures, lockdowns/movement restrictions, and GBV protections adopted by states in their COVID-19 response from March to August 2020. We reviewed emergency orders and other official guidance issued during this period related to non-essential business closures, essential service designations, and movement restrictions, including stay-at-home orders. We cross-checked our analyses with another state policy review of GBV protections during the COVID-19 response [33]. We defined GBV protections as any statewide policies, orders, or official guidance exempting GBV survivors from movement restrictions, thereby protecting their right to leave their homes and seek shelter or other services.

The survey targeted 519 GBV and sexual and reproductive health service providers across ten states, selected as a purposive sample for the survey to reflect the diversity of state environments during this period, considering various factors that converged to potentially influence access to, and provision of, GBV services during the pandemic. Starting with a broad list, states were narrowed with an intention to include a range of geographic regions, demographic compositions, and policy landscapes, informed by findings from the state-by-state emergency policy review. We included states with COVID-19 hotspots or concurrent racial justice movement epicenters, that is, states that experienced significant mass protests and civil unrest in the wake of George Floyd’s murder in May of 2020, to examine the converging effects of both events on service providers. The online Qualtrics survey was distributed through email contact information for service providers found through a web-based search and through existing listservs related to GBV prevention and response. The survey included 39 questions and required a maximum of 20 minutes for completion. Questions included respondents’ primary role at the organization, how COVID-19 has impacted GBV service provision, the reasons GBV services stopped or were reduced, workload increases, and underserved groups. The final sample included 77 GBV service providers.

For qualitative semi-structured interviews, we conducted in-depth interviews with eleven GBV service providers from six states. Respondents served a variety of populations, with a focus on providers serving historically underserved populations (including Black, Indigenous, and People of Color, immigrants, LGBTQ individuals, and incarcerated populations) who may be most affected by service disruptions. Interviewees spanned a range of organizations, including direct service providers, funders, and organizations conducting advocacy and policy work. Service providers were identified through web-based searches and expert recommendations and contacted via e-mail. Study investigators developed an English interview guide focused on the impact of COVID-19 and resulting policies on the availability and modality of GBV services, facilitators and barriers to service provision, and government responses to GBV challenges during the pandemic. Interviews were conducted over Zoom or telephone per respondent preference by two members of the research team and lasted approximately 45–60 minutes. All interviews took place between August and October of 2020. Interviews were audio-recorded with participant consent and subsequently transcribed by the interviewers. Two members of the study team independently coded the transcripts line-by-line by hand and conducted thematic analysis to identify key, cross-cutting themes using both inductive and deductive analysis. Three major cross-cutting themes emerged: reductions in GBV service provision and quality and increased workloads, shifts in service utilization, and funding impacts.

Given that states were purposively selected based on social and political factors relevant to GBV care to explore the role these factors played in service provision, the findings are only representative of those included in the sample. Study procedures were approved by Columbia IRB (IRB-AAAS9211). Oral informed consent was obtained for all interview participants at the start of the Zoom or phone session and witnessed by the study team member conducting the interview. Electronic informed consent was obtained for all survey participants at the beginning of the online survey. Data were stored and protected per IRB guidelines.

## Results

### Policy review

**Fig 1:**
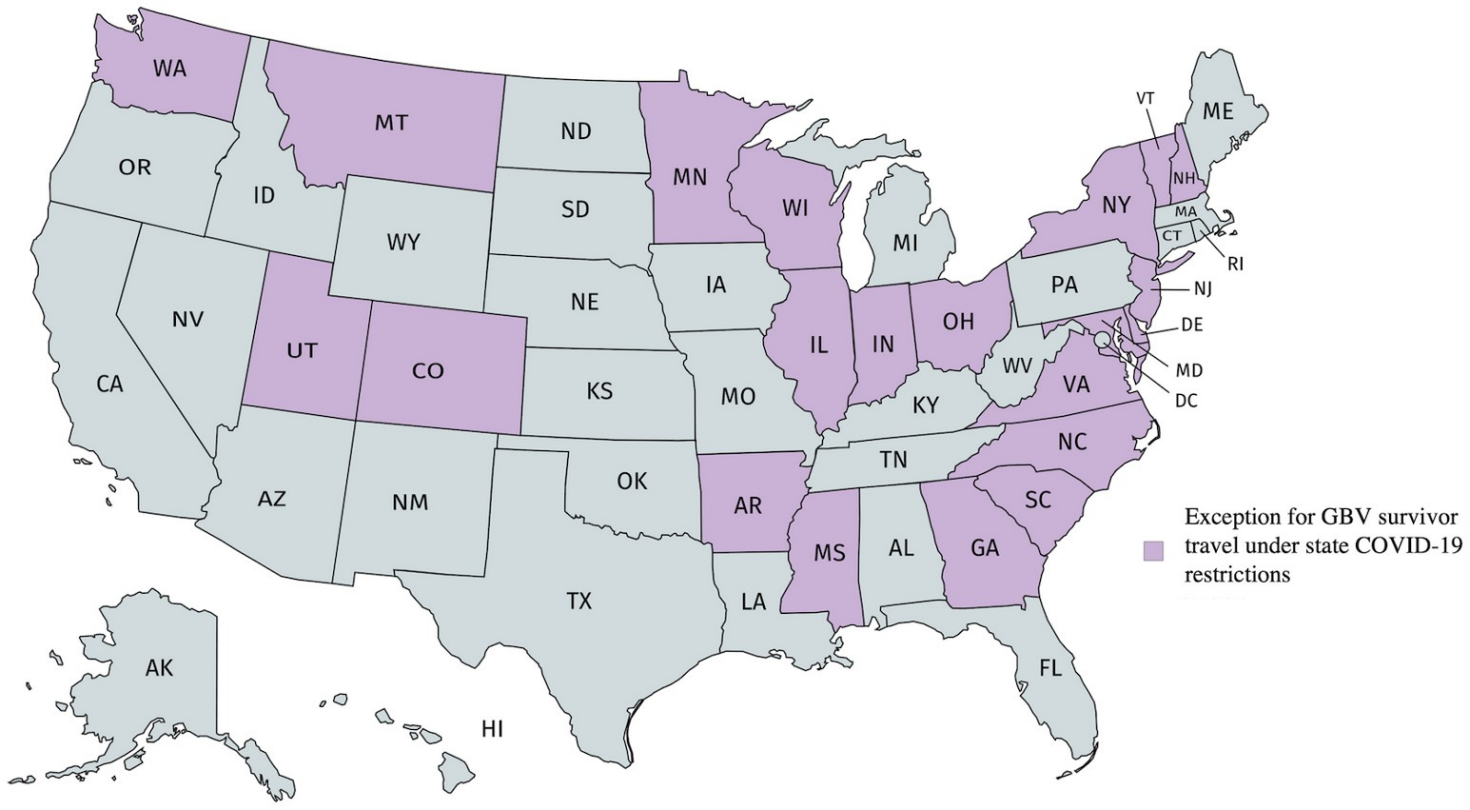
U.S. states that exempted some or all GBV survivors from COVID-19 emergency measures. Republished from MapChart.net under a CC BY license, with permission from MapChart, original copyright 2022.

In March and April of 2020, all states issued emergency declarations related to COVID-19, 45 states closed “non-essential businesses,” and 44 states issued lockdowns/movement restrictions, including orders to stay home [34]. Twenty-one states enacted protections for GBV survivors (Fig 1). Precise protections varied by state, but many focused on exempting domestic violence survivors from movement restrictions, allowing people experiencing or at risk of experiencing GBV to leave their places of residence and seek safety and/or services. For example, Washington’s stay-at-home order stated that “This prohibition shall not apply to individuals whose homes or residences are unsafe or become unsafe, such as victims of domestic violence” and urged people experiencing violence “to leave their homes or residences and stay at a safe alternate location” [35]. Five of the states classified as having GBV protections did not reference domestic violence specifically, and instead included blanket exemptions to movement restrictions for all people experiencing safety concerns in their place of residence. For example, the New Jersey governor issued an order for all residents to stay at home unless “leaving because of a reasonable fear for his or her health or safety” [36]. A few states established exceptions to movement restrictions outside of stay-at-home orders. For example, Arkansas never issued a stay-at-home order, but its emergency declaration explicitly exempts survivors of domestic violence from restrictions on staying in short-term rentals and commercial lodging during the COVID-19 pandemic [37].

Protections varied somewhat with time, with more states adding or updating protections after issuing their first series of emergency orders. Some states, like Minnesota, included exemptions for survivors in their first iteration of pandemic emergency orders [38]. Others added protections in new, stand-alone emergency orders, such as New Hampshire’s order to establish a COVID-19 emergency relief fund for domestic and sexual violence services [39]. A few states, such as New York, amended emergency orders to include or clarify references to exemptions from movement restrictions for people experiencing domestic violence or safety concerns [40]. Twenty-nine states did not enact protections for GBV survivors as of July 2020. (See S1 Table for a full list of U.S. states categorized by GBV protections related to mobility restrictions).

Protections related to nonessential service closures intersected with shifting federal guidance. In orders to close non-essential businesses, five states included explicit exemptions for GBV service providers, such as domestic violence shelter staff [33]. At the federal level, the Cybersecurity and Infrastructure Security Agency (CISA) published an “Advisory List” intended to help state, local, and territorial officials define their essential workforce. The third version of the guideline, issued on April 17^th^, exempted essential workers from shelter-in-place orders, curfews and other movement restrictions [41]. The fourth version, issued on August 18^th^—about five months after the initial emergency declarations—was the first to clearly establish GBV service providers as covered by this exemption, stating that movement restrictions should not apply to “Workers who provide human services… case managers, crisis counselors, adult protective services personnel, child protective services personnel, domestic violence counselors, human trafficking and recovery personnel” [42].

### Survey of GBV service providers

A total of 77 GBV service providers completed the survey (Table 1). Of those respondents, the majority (70%) provided only GBV services. Nearly half (45%) were employed at community-based organizations, and nearly one-third (35%) worked at non-governmental organizations. Survey results indicate reductions in service provision, shifts in service utilization, changes in GBV reporting, and exacerbation of GBV inequities for underserved groups.

**Table 1:** Descriptive characteristics, 2020 US GBV service providers survey (N = 77).

	GBV Service Providers (N = 77)	
Key Variables	N	%
**Area of Work**		
GBV	54	70%
GBV & SRH (Sexual and Reproductive Health)	23	30%
**Type of Organization**		
NGO (Non-Governmental Organization)	27	35%
CBO (Community Based Organization)	35	45%
Shelter	21	27%
Other (International Organization, Government, Health Facility)	14	18%
**Respondents Primary Role**		
Program manager	26	34%
Health or Social worker	16	21%
Community Health Worker/Educator or Trainer	9	12%
Other	26	34%
**COVID-19 has Impacted GBV**	45	58%
**How COVID-19 has impacted GBV (N = 45)**		
Reports of GBV/IPV increased	25	56%
Reports of GBV/IPV reduced	4	9%
Reports are mixed*	9	20%
**GBV service disrupted due to COVID-19** **		
Clinical management of rape or other GBV	9	12%
Counselling or psychosocial services	13	17%
Shelter and/or other social services	7	9%
GBV case management services	10	13%
Community-based GBV prevention/awareness activities	38	49%
Legal support for GBV survivors	22	29%
**Reason GBV Services Stopped/Reduced**		
Deemed non-essential	6	8%
Lockdown/Movement Restrictions	40	52%
Limited Supplies/Commodities	8	10%
Insufficient Personal Protective Gear	6	8%
Staff or funding diverted to Emergency Response	6	8%
Demand for services reduced	15	19%
Remote capacities are limited	19	25%
**Workload Increases**		
Added Responsibility of Emergency Response	16	21%
Demand for Services Increased	34	44%
**Underserved Groups**		
Adolescents	21	27%
Women with disabilities	24	31%
Black Indigenous People of Color	29	38%
Migrants, refugees, displaced people	32	42%
LGBTQ	15	19%

Note: Categories are not mutually exclusive.

*Some forms of GBV (such as IPV) have increased, but other forms (such as non-partner violence) have reduced.

**Disruptions include services stopped completely, or services stopped, but are not either partially or fully available.

All participants (100%) agreed their work had been impacted by COVID-19. Over half (58%) thought COVID-19 had impacted the prevalence of GBV in the U.S., of which 56% of respondents noted an increase in GBV reporting and 20% noted an increase of some forms of GBV (such as intimate partner violence), but a reduction in other forms of GBV (such as non-partner violence). The most frequent GBV service disruptions were for community-based GBV prevention or awareness-raising activities (49%), legal support for GBV survivors (29%), or counselling or psychosocial services (17%). Over half of all respondents (52%) reported their GBV work stopped or was reduced due to lockdowns/movement restrictions at the beginning of the pandemic, and a quarter (25%) reported limited capacity to provide remote services.

Findings indicate that GBV work stopped or was reduced due to a reduction in demand (19%). However, staff also reported a simultaneous increase in workload due to increased service demand (44%) and to support the emergency response in addition to regular work (21%). Over half of respondents (52%) reported developing innovative ways to continue service delivery using technology and other strategies. Survey respondents also identified groups underserved in the first six months of pandemic response including adolescents (27%), women with disabilities (31%), Black, Indigenous, and People of Color (38%), migrants, refugees, and displaced populations (42%), and LGBTQ individuals (19%).

### In-depth interviews

Eleven GBV organizations working in six states participated in interviews (Table 2). Most interviewees worked for direct service organizations (82%) and held leadership roles (73%). Three major cross-cutting themes surrounding COVID-19’s impact on GBV services emerged from the interviews: reductions in GBV service provision and quality and increased workload, shifts in service utilization, and funding impacts.

**Table 2:** Selected characteristics of GBV service provider, advocate, and funder interviewees, United States, 2020 (N = 11).

Characteristic	N	Frequency (%)
**Organization’s Primary Role**		
Direct Services	9	82%
Advocacy/Policy	1	9%
Funder	1	9%
**Interviewee Role**		
Leader^1^	8	73%
Program Staff^2^	3	27%
**Region**		
Midwest	7	64%
South	3	27%
Northeast	1	9%
**Funding Sources** ^3^		
Individual Donors	11	100%
Foundations	10	91%
Government Grants	**7**	64%

^1^ Director, president, founder, or other high-level leadership role.

^2^ Direct program implementation role, without supervisory duties.

^3^ Six interviewees had all three types of funding.

#### Reductions in GBV service provision and quality and increased workloads

Respondents described significant operational disruptions resulting in reduced provision of certain GBV services and diminished service quality. Providers described closing facilities and reducing capacity; struggling to implement public health recommendations; shifting to virtual service provision; adapting to the disruption of key partnerships; and navigating civil unrest as protests against racial injustice layered alongside the pandemic.

In an effort to limit the spread of disease and mitigate fear of infection, shelters temporarily closed, reduced capacity, or restricted who they allowed to stay given perceived risk of disease transmission. This aligns with survey respondents who also indicated that closures of GBV projects and programs were common. Facilities that remained open were forced to navigate myriad challenges to keep clients and staff safe, such as increasing cleaning and sanitation, staggering eating times, enforcing masking and distancing, or moving in-house therapy visits online. These added duties may have contributed to increased workloads, as supported by the survey results.

Key partnerships were also disrupted. Hospital restrictions meant GBV advocates were no longer allowed into emergency rooms for sexual assault exams. Interruptions to in-person client access complicated and delayed the provision of time-sensitive support services, as detailed by one respondent:

*“*.*…either we saw less people who would present at the ER requesting over*-*the*-*phone advocacy*, *so that would affect the continued advocacy we would be able to provide*, *or…the paperwork wouldn*’*t get where it needed to go in time*, *so that person might have to wait two weeks before getting a follow*-*up phone call*.*”*

– R11, Service provider, South

Virtual platforms allowed some advocacy, case management, counseling services, and court hearings to continue. However, the adjustment was described as slow and cumbersome. Respondents pointed out that the transition to virtual platforms limited the reach of remaining programs to people with internet access, deepening the digital divide. Many respondents also felt remote services were less effective because they lacked a personal, emotional connection and described the human presence as critical to GBV service provision. In some cases, the switch to virtual work simply did not occur. One respondent described how local coordination meetings completely stopped, further fragmenting the response by interrupting the pre-pandemic framework for collaboration among local agencies.

Service disruptions due to the pandemic layered alongside civil unrest as protests unfolded across the U.S. following the murder of George Floyd on May 25th, 2020. Interviewees with facilities close to protest epicenters expressed safety concerns for shelter residents and staff:

*“We had about a 7*-*day period right after the murder of George Floyd where we moved everybody who was in the shelter to a separate safe location*…*there was a lot of street violence and unrest immediately after that*, *so we took families and advocates out of the structure for about a week*.*”*

– R3, Service Provider, Midwest

The movement to address racial injustice added another dimension to GBV service providers’ shifting workloads. Some respondents viewed engagement in the racial justice movement as integral to addressing gender-based violence, especially in relation to long-term efforts. These respondents participated in protests as a means of demonstrating solidarity, increasing outreach, and distributing resources. One respondent discussed how their organization was accommodating staff needs and revamping priorities in response to the intersecting crises of COVID-19 and structural racism:

*“*…*what has impacted our work more has been the racial violence and unrest*…*we*’*ve had to institute self*-*care days*…*we pay a therapist for some of our staff members to support the movement and to support Black lives*. *Part of our work has changed in that white and other POC folks have taken on more*. *So that community*, *they*’*re not off*. *They*’*re out in the Black community organizing*, *helping get mutual aid to people in crisis*.*”*

– R8, Service Provider, Midwest

#### Shifts in service utilization

Respondents described both increases and decreases in GBV service uptake. Service providers’ explanations for these changes often related to restrictions under emergency orders. Interviewees also described how the pandemic overlaid existing inequities in access to GBV services among historically oppressed groups, contributing to ongoing disparities in service utilization.

Several organizations reported a decrease in clients following statewide emergency orders. Some respondents attributed this to an assumption that GBV service providers were closed due to orders to stay home and close non-essential business even if providers never actually closed their doors. Other respondents cited people’s restricted autonomy under stay-at-home orders, in addition to fear of virus exposure, as possible explanations for early reductions in service uptake, which were followed by an increase when restrictions were relaxed:

*“I think a lot of people were stuck with their abusers*, *so they didn*’*t have means to access our services*, *and now that the safer*-*at*-*home order has lifted*, *our crisis line has been ringing nonstop*… *But I still think there have been people who have been stuck at home because they*’*re afraid of the virus and their abuser*….*it*’*s one thing to be afraid of the person you live with*, *but then to also be afraid of leaving the house because you catch a deadly disease…that*’*s very hard*.*”*

– R7, Service Provider, Midwest

Service providers’ association of emergency restrictions with decreased utilization of GBV services paralleled responses from survey respondents, the majority of whom indicated lockdowns/movement restrictions as reasons for GBV service disruptions. While some GBV organizations initially had to do renewed outreach to reassure people their services were still operating, others experienced a surge in requests for support early in the pandemic:

*“In the first six weeks*, *we got 150 calls*. *We had had that amount in a year…we were getting a lot of calls from…queer and trans college age students*, *and high schoolers who were now stuck at home with their abusers*, *that being their parents*. *And that was very different than intimate partner violence that we had known so well*. *This was like*, *this is a pandemic and this is causing family violence to escalate*. *And then we started to hear from more trans people who were homeless and had nowhere to go and didn*’*t have good experiences in shelters*.

– R8, Service Provider, Midwest

Even though demand increased in some cases, respondents stated that access to GBV services remained unequal. They identified several groups with less access to GBV services prior to the pandemic:

*“*…*people of color*, *queer and trans people*, *anybody that*’*s outside of*, *you know*, *the perfect victim sort of image of white*, *middle class*, *able*-*bodied Christian*, *etc*. *etc*.*”*

– R21, Service Provider, Northeast

Shifts in GBV prevalence during the pandemic interacted with existing policies and practices that had diminished availability and access to GBV services for historically oppressed groups. Similar to survey respondents’ identification of migrants as underserved, a respondent working at the U.S.-Mexico border described how immigration enforcement limited access to services among migrant women:

*“We*’*ve…seen a number of sexual assaults that occurred in the United States*, *that the individual tried to file a criminal complaint*. *And then when it was discovered they were undocumented*, *their criminal complaint was never taken and they were instead just deported*.*”*

– R15, Service Provider, South

The intensification of social isolation experienced by historically oppressed groups during the pandemic emerged as another possible explanation for deepening inequities in access to services. One interviewee drew on refugees’ experiences to illustrate how layers of isolation sustained barriers to access:

*“The populations we work with are typically isolated a few times over*. *So I would say the thing that COVID has exacerbated is definitely the isolation of*, *an example would be a recent refugee family where there*’*s low English proficiency because they*’*ve only been here 6 or 12 months*, *and if the husband is very controlling and is perpetrating abuse*…*and just not allowing the woman to even understand what resources are available in the community*.*”*

– R3, Service Provider, Midwest

#### Funding impacts

Respondents discussed a range of ways the COVID-19 pandemic changed the GBV funding landscape. Like other activities, fundraisers shifted to a virtual format. Interviewees described grappling with unexpected COVID-related costs. Their comments suggested an emerging tension between appreciation for emergency funding opportunities and concern about the long-term availability of funding. As with service utilization, providers’ comments indicated that disparities in resources and funding were exacerbated during the pandemic.

Immediate impacts included funding organizations being forced to close or service providers having to move fundraisers online. For one respondent, this was especially disappointing given the organization’s focus on grassroots fundraising given federal contracts are not sufficient to cover their expenses or effect major social change. Some respondents explained that their expenses had increased to cover COVID-related costs, such as purchasing PPE or filling service gaps when partners closed:

*“The amount of work we have has nearly tripled*. *The amount of financial need we have has nearly tripled in the last few months since COVID started*. *And the amount of funding coming through has decreased because of COVID*.*”*

– R4, Service Provider, South

To cover unexpected costs, interviewees mentioned taking advantage of emergency funding opportunities. These funds were not always specifically earmarked for GBV service provision. Despite this short-term influx of resources, many organizations expressed uncertainty about future funding and noted the emergency funds would run out quickly given their current needs. Interviewees generally did not describe specific situations in which they were forced to close or reduce services due to the diversion of funding to other aspects of the COVID-19 response. In some cases, continued operations were supported by funders increasing their flexibility to give grantees more autonomy over resource allocation:

*“They allowed us to move more money over that we weren*’*t going to spend on other things*…*like we*’*re not spending money on travel because we*’*re not going anywhere*, *so we*’*re able to put that into transportation costs for survivors*. *So there*’*s been more flexibility in budgets… to be able to do as much as we can for both staff and survivors*.*”*

– R21, Service Provider, Northeast

Lack of flexibility among funders contributed to organizations’ concerns about long-term funding. One respondent with inflexible funders expressed fears of being stuck with unfunded expenses for PPE and other new costs while facing potentially smaller funding in future years because they had not fully spent their travel and in-person client services budgets.

Perceptions of funder priorities constrained some organizations from engaging in new partnerships and opportunities for intersectional work during the pandemic. One respondent described refraining from pursuing intersections with sexual and reproductive health services and access to abortion:

*“I feel like the Board of Directors and even our Executive Director is hesitant to be vocal about certain topics*, *because they are concerned about what the conservative community would think and how it would affect funding*.*”*

– R11, Service Provider, South

Other respondents described perceptions of funder priorities as limiting their organizations’ involvement in racial justice work, including the Black Lives Matter (BLM) movement over fear of donors pulling funds. Another respondent stated that funder restrictions prohibited them from offering services to incarcerated and formerly incarcerated individuals:

*“*…*one of the problematic things about our funding is that we cannot work with people who are considered* ’*offenders*’…*we believe in restorative and transformative justice*, *but we actually can*’*t do that work*…*because of the funder restrictions that we serve victims only*.*”*

– R8, Service Provider, Midwest

The tendency of funder priorities to limit engagement in intersectional work and bar certain groups from accessing services overlapped with pre-pandemic frameworks for resource allocation to further exacerbate disparities in access to GBV services. As a result, unmet needs for GBV services increased among historically underfunded and underserved groups. One interviewee elaborated on the deepening disconnect between needs and resources:

*“*…*resources are really allocated almost by zip codes*. *And the pandemic has really reinforced that… So low*-*income and communities of color*, *specifically Indigenous though*. *And I think that the new influx of immigration women are really out of the loop*.*”*

– R18, Service Provider, Midwest

## Discussion

The COVID-19 policy response led to reductions in GBV service provision and quality and increased workloads, shifts in service utilization, and exacerbations of funding gaps. These disruptions overlaid long-term policy and funding limitations, disproportionately burdening historically oppressed and underserved groups such as migrants, refugees, and displaced people, Black, Indigenous, and People of Color, women with disabilities, adolescents, and LGBTQ individuals. Prior research has documented that emergencies are associated with increases in GBV globally [43, 44] and that access to GBV health services are critical to improving health outcomes [16–18]. Since the onset of the COVID-19 crisis, numerous commentaries [21, 27–29] and a limited number of studies, but none yet in the U.S., [24, 26, 30–32] have indicated an uptick in GBV and disruption of service provision during the pandemic. This study expands on this knowledge by investigating the specific ways in which COVID-19 responses altered GBV health service provision in the U.S.

Only five states included explicit exemptions for GBV service providers in Orders to close non-essential businesses. The federal government did not issue guidance to include GBV service providers in essential worker exemptions until five months after the initial emergency declaration. This aligns with much of what was happening in low-, middle-, and high-income countries around the world, with very few countries prioritizing GBV services initially [24, 30, 45–47]. Both survey and interview findings indicate that lockdown and/or movement restrictions stopped or reduced service provision. Respondents were meeting an increase in client demand while also tending to new requirements, including supporting the emergency response, developing new hygiene protocols to mitigate COVID-19 transmission, building and implementing virtual services, and participating in and building synergies with concurrent racial justice movements.

While the policy review found twenty-one states eventually enacted protections for GBV survivors, some months after initial restrictions, twenty-nine states did not. Survey and interview findings indicate the fragmented policy landscape led to client confusion regarding which services were open, exacerbated by the fact that community-based GBV prevention or awareness-raising activities were often forced to halt, and restrictions disrupted referral pathways and partnerships. This disruption is consistent with studies and reviews from Kenya and Uganda, where facilities saw a decrease in clients due to limited outreach informing the community of service availability and the disruption of normal referral pathways [24, 46, 47]. Findings also indicate that respondents adopted new virtual platforms for service delivery, but these platforms challenged the provider-client relationship due to strained personal connection building and confidentiality concerns, as was similarly documented in the switch to virtual tools for GBV health services in the United Kingdom [47]. Virtual tools can deepen disparities in access to services for people who lack access to the Internet, smartphones, and/or computers. This is in contrast to other findings on the use of virtual tools for medication abortion, which has been shown to improve service utilization and reduce inequities in access posed by financial hardship and child care or work responsibilities [48]. GBV services may be unique among other gender-based and sexual and reproductive health (SRH) services in the increased importance of in-person care provision.

In line with other findings, COVID-19 had an impact on GBV service utilization [23, 47]. The multiple service utilization changes were noted in survey responses and expanded upon in in-depth interviews. These included a perceived increase in GBV, a reduction in service utilization at certain timepoints during the pandemic (for example due to restricted autonomy under stay-at-home orders leaving people stuck with their abusers), and a change in the needs of clients who did access services (for example, increased reported needs for family violence services). Several commentaries and studies have reported similar findings, including an uptick in intimate partner violence and the number of minors presenting for care during the pandemic in Kenya, due to increased time spent at home as a result of school and business closures [47], a perceived increase in violence but reduction in reporting in Ecuador [49] and Brazil [50], and an increase in first time violence in Australia [32]. Preparing health services for service utilization changes is thus an essential element of GBV response in emergencies.

Funding emerged as a key challenge. Even prior to the pandemic, federal funding did not meet GBV service providers’ needs, and COVID-19 exacerbated these challenges. Respondents reported service disruptions due to limited resources and that expenses grew due to efforts to reduce COVID-19 transmission, including PPE, extra cleaning, and distancing requirements. Some providers were able to take advantage of emergency funding, while competing resources for other emergency COVID-19 response during the pandemic emerged as a key challenge in other reports [24, 47]. Inflexible funding and donor restrictions aggravated care siloes by shaping providers’ willingness and ability to provide holistic sexual and reproductive health care, often a need of GBV clients, to participate in the Black Lives Matter movement, or to offer services to incarcerated and formerly incarcerated individuals due to fear of donor backlash and/or denial of funds.

The changing nature of GBV prevalence and service provision during the pandemic interacted with existing policies and funding opportunities that limited GBV service utilization for historically oppressed groups in this study and others [24, 26]. Access to GBV service utilization remained unequal during the pandemic particularly for migrants, refugees, and displaced people, Black, Indigenous, and People of Color, women with disabilities, adolescents, and LGBTQ individuals. The burden of the crisis on historically oppressed groups was consistent with other reports that found Black, migrant, Trans, Indigenous, and Roma women were particularly affected by GBV amid COVID-19 in Latin America [51], as were Black, low-income women in South Africa [45]. Addressing the impact of COVID-19 and GBV on historically oppressed groups through programmatic, policy, and funding efforts is thus an important undertaking.

### Limitations

Despite being the first study in the U.S., and one of the first in the global north, to document the impact of COVID-19 on GBV service delivery, there are limitations. Although service providers from all four U.S. geographic regions were invited to participate, the majority (64%) of qualitative interview participants came from the Midwest, limiting the generalizability of these interviews to regions with different policy landscapes. As with all qualitative work, these findings should be interpreted as a reflection of included service provider experiences. In addition, findings were collected early in the pandemic. More time may change the nature of some of these challenges, including, for example, virtual services’ evolving capabilities and utility in GBV service provision and the effect of COVID-19 on funding for GBV health services. Future research is needed to assess these long-term effects.

## Conclusion

Federal laws and regulations that chronically underfund and silo GBV prevention and response from broader health services left U.S. GBV service providers unprepared for the scale of COVID-19 challenges faced during the pandemic. Both federal and state governments’ initial failure to prioritize GBV care in their COVID-19 responses added stressors to an existing political, economic, and social landscape that created and sustained inequities and inefficiencies in GBV service provision as found in the integrated policy review, survey, and interview results. These inequities and inefficiencies challenged GBV providers’ abilities to address changing demands and operating contexts. Understanding these impacts may encourage policymakers, service providers, and advocacy groups to better prioritize GBV health services in future emergencies and strengthen GBV service delivery by addressing barriers to care and the exclusion of underserved populations. Future policies, in emergency and non-emergency contexts, should recognize that GBV is essential care and ensure comprehensive services for all clients, particularly members of historically oppressed groups. Emergency preparedness plans should include clear policy guidelines that deem GBV health services essential, outline communication plans to inform survivors of service availability, and address the needs of historically oppressed populations to ensure equitable service utilization through improved service access to address inequities.

## Supporting information

S1 Table List of U.S. states categorized by GBV protections related to mobility restrictions (March–July 2020). (DOCX)

S1 File Qualtrics survey. (DOCX)

S2 File In-depth interview guide. (DOCX)

S3 File MapChart copyright permissions. (PDF)
