# Supplementary material for: COVID-19 and gender-based violence service provision in the United States
Source: PLoS One. 2022 Feb 16;17(2):e0263970. doi: 10.1371/journal.pone.0263970 (PMC8849472; doi:10.1371/journal.pone.0263970)
Supplement: S1 Table — (DOCX) [file pone.0263970.s001.docx]

**S1 Table**. **List of US states categorized by GBV protections related to mobility restrictions (March-July 2020).**

| **Explicitly exempt survivors of domestic violence from mobility restrictions** | **Exempt individuals with safety concerns in general from mobility restrictions** | **No GBV-related exemptions to mobility restrictions** |
| --- | --- | --- |
| Arkansas^1^  Colorado  Delaware  Illinois  Indiana  Maryland  Minnesota  Mississippi  Montana  New Hampshire  New York  North Carolina  Ohio  Utah  Washington  Wisconsin | Georgia  New Jersey  South Carolina  Vermont  Virginia | Alabama  Alaska  Arizona  California  Connecticut  Florida  Hawaii  Idaho  Iowa^2^  Kansas  Kentucky  Louisiana  Maine  Massachusetts  Michigan  Missouri  Nebraska^2^  Nevada  New Mexico  North Dakota^2^  Oklahoma  Oregon  Pennsylvania  Rhode Island  South Dakota^2^  Tennessee  Texas^2^  West Virginia  Wyoming^2^ |

^1^ No statewide stay-at-home order in place; exemption facilitates movement to a safe space by protecting access to short-term lodging for GBV survivors

^2^ No statewide mobility restriction, such as a stay-at-home order, in place
