# Supplementary material for: COVID-19 and gender-based violence service provision in the United States
Source: PLoS One. 2022 Feb 16;17(2):e0263970. doi: 10.1371/journal.pone.0263970 (PMC8849472; doi:10.1371/journal.pone.0263970)
Supplement: S1 File — (DOCX) [file pone.0263970.s002.docx]

**S1 File. Qualtrics survey.**

| S.NO | Items | Response Categories | Skips |
| --- | --- | --- | --- |
| 1 | Which country are you working in? | 1 Colombia  2 Kenya  3 Nigeria  4 South Africa  5 Uganda  6 USA  7 Other **[Please elaborate]** | 🡪 2 |
| 2 | Which state are you working in | Which state are you working in?   1. Louisiana 2. Maine 3. Minnesota 4. New Mexico 5. New York 6. North Carolina 7. South Dakota 8. Texas 9. Washington 10. Wisconsin |  |
| 3 | What kind of organization do you work for?  **Please select all that apply** | 1. Local NGO 2. International NGO 3. International Organization (for example, UN agencies, World Bank, etc) 4. Government 5. Community-based Organization 6. Health facility (for example, Clinic, health center, hospital, etc) 7. Shelter 8. Other **[Please elaborate]** |  |
| 4 | What is your area of work? | 1 Gender-based violence (GBV)  2 Sexual and reproductive health (SRH)  3 Both GBV and SRH  4 We do not work on either GBV or SRH | 🡪 Exit |
| 5 | What age group(s) does your organization serve?  **Please select all that apply** | 1. Children (0-9 years) 2. Very young adolescents (10-14 years) 3. Adolescents (15-17 years) 4. Adults (18 years or older) 5. Any age |  |
| 6 | How would you best describe your primary role? | 1. Program manager 2. Health worker (for example, nurse, midwife, doctor, etc) 3. Social worker 4. Community outreach worker / Community health worker 5. Educator/Trainer 6. Other **[Please elaborate]** |  |
| 7 | Has COVID-19 impacted your work or changed how you work? | 1. Yes   0. No | 🡪 14 |
| 8 | How has COVID-19 impacted your work at any point since the pandemic began?  **Please select all that apply** | 1. Work has stopped/reduced as SRH and/or GBV clinics/services were deemed non-essential by the government 2. Work was stopped/reduced due to lockdown/movement restrictions that have been imposed since the beginning of the pandemic 3. Work was stopped initially due to lockdown/movement restrictions imposed in the beginning of the pandemic, but have since resumed after restrictions were reduced/lifted 4. Work has stopped/reduced as supplies and commodities are unavailable or limited in supply (for example, due to difficulties with procurement and transport) 5. Work has stopped/reduced as we do not have sufficient personal protective gear and other infection management supplies 6. Work has stopped/reduced as our staff have been diverted to emergency response 7. Work has stopped/reduced as our funding was diverted to emergency response 8. Work has stopped/reduced as we do not have the capacity and resources to switch to remote work 9. Work has reduced as use or demand for services has reduced 10. Work has reduced as only some work can be done remotely 11. Workload has increased as we are being asked to support the emergency response in addition to our regular work 12. Workload has increased as use and demand for services has increased 13. We are trying innovative ways to continue service delivery using technology and/or other strategies 14. Other **[Please elaborate]** |  |
| 9 | What gender-based violence (GBV) services does your organization provide and which ones have you limited or stopped since the beginning* of COVID-19?  **Please select all that apply**  **( * by beginning we mean when lockdowns and other restrictive policies were imposed)** | ***Service provision unchanged from beginning (1) Limited service provided from beginning (2) Service stopped completely from beginning (3) Services stopped initially but full services available now (4) Services stopped initially but limited services available now (5) Not applicable/ we do not provide this service (6)***     1. Clinical management of rape or other GBV 2. Counselling or psychosocial services 3. Shelter and/or other social services 4. GBV case management services 5. Community-based GBV prevention/GBV awareness-raising activities 6. Legal support for GBV survivors 7. Other (Please elaborate) |  |
| 10 | What sexual and reproductive health (SRH) services does your organization provide and which ones have you limited or stopped since the beginning* of COVID-19?  **Please select all that apply**  **( * by beginning we mean when lockdowns and other restrictive policies were imposed)** | ***Service provision unchanged from beginning (1) Limited service provided from beginning (2) Service stopped completely from beginning (3) Services stopped initially but full services available now (4) Services stopped initially but limited services available now (5) Not applicable/ we do not provide this service (6)***  1. Contraceptive counselling and services  2. Abortion care/post abortion care  3. Delivery care  4. Antenatal/postnatal care  5. STI screening and treatment  6. HIV testing, care and treatment  7. Reproductive cancer screening   1. Adolescent-friendly or focused SRH services 2. Other SRH Clinical services (Please elaborate) 3. Community outreach and awareness related to SRH 4. Other (Please elaborate) |  |
| 11 | Do you see that any particular groups of women are having more trouble accessing your GBV or SRH services or are accessing these services less during the pandemic? | 1. Yes   0. No  8. Don’t know | 🡪14  🡪14 |
| 12 | Which groups of women are having more trouble accessing your GBV or SRH services or accessing them less during the pandemic? **Please select all that apply** | 1. Adolescents 2. Women with disabilities 3. Ethnic minorities 4. Migrants, refugees or other displaced people 5. People of diverse sexual orientation, gender identity and expression or sex characteristics 6. Unmarried women 7. Other **[Please elaborate]** |  |
| 13 | Has your organization utilized any innovative mechanism to fill some of the gaps in GBV/SRH services and prevention efforts? | 1 Yes  0 No  8 Don’t know | 🡪15  🡪15 |
| 14 | Please provide an example of how your organization has utilized innovative mechanisms to fill some of the gaps in GBV/SRH services and prevention efforts. |  |  |
| 15 | Are you or members of your organization involved in any local, regional or national taskforce on COVID-19 response and recovery? | 1 Yes  0 No  8 Don’t Know |  |
| 16 | To support your COVID-19 (GBV and/or SRH) response has your organization received new or additional funding from:  **Please select all that apply** | YES (1) NO (0) NA (9)   1. Bi-lateral donors 2. Multi-lateral donors 3. National Government 4. Private foundations 5. Community/private donations 6. Other (Please elaborate) |  |
| 17 | As a result of COVID-19 has (GBV and/or SRH) funding to your organization been reduced or stopped from:  **Please select all that apply** | YES (1) NO (0) NA (9)   1. Bi-lateral donors 2. Multi-lateral donors 3. Private foundations 4. National Government 5. Community/private donations 6. Other (Please elaborate) |  |
| 18 | What else are current funders doing to support your organization’s effort to respond to the impact of COVID-19?  **Please select all that apply** | YES (1) NO (0)   1. Converting restricted grant to unrestricted funding 2. Accelerating payment schedules on grants 3. Extending the timeframe of current grant(s) without penalty 4. Allowing goals of current grant(s) to shift 5. Waiving or making reporting deadlines flexible 6. Communicating one-on-one with you about the effect of   COVID-19 on your organization   1. Other (Please elaborate) |  |
|  | **THE FOLLOWING QUESTIONS PERTAIN TO GENERAL GBV AND SRH SERVICE PROVISION IN THIS COUNTRY (NOT LIMITED TO YOUR ORGANIZATION)** | |  |
| 19 | Has COVID-19 impacted GBV service provision and prevention in this country? | 1 Yes  0 No  8 I don’t know/ not my area of expertise | 🡪24  🡪29 |
| 20 | How has COVID-19 impacted GBV service provision and prevention?  **Please select all that apply** | 1 Full on-site services are available but there has been a reduction in use or demand  2 Full on-site services are available but there has been an increase in use or demand  3 Services have stopped as GBV clinics/services were deemed non-essential by the government  4. Services have stopped/reduced since the beginning of the pandemic due to lockdown/movement restrictions imposed in the country  5. Services were stopped initially due to lockdown/movement restrictions imposed in the beginning of the pandemic, but have since resumed after restrictions were reduced/lifted  6. Services have stopped/reduced as staff have been diverted to emergency response  7.services have stopped/reduced as funding has been diverted to emergency response    8. Services have stopped/reduced as there are shortages of personal protective gear and infection management supplies  9. Services have been reduced as use or demand has decreased  10. Services have reduced as staff have additional COVID-19 responsibilities  11. Services have reduced as essential supplies and commodities are in short supply (for example, due to difficulties with procurement and transport)  12 Service delivery has shifted to remote provision via technology or other innovative strategies  13 Other **[Please elaborate]** |  |
| 21 | What type of GBV services have been limited or stopped since the beginning* of COVID-19?  **Please select all that apply**  **( * by beginning we mean when lockdowns or other restrictive policies were imposed)**? | ***Service provision unchanged from beginning (1) Limited service provided from beginning (2) Service stopped completely from beginning (3) Services stopped initially but full services available now (4) Services stopped initially but limited services available now (5) Not applicable/ we do not provide this service (6)***   1. Clinical management of rape or other GBV 2. Counselling or psychosocial services 3. Shelter and/or other social services 4. GBV case management services 5. Community-based GBV prevention/GBV awareness-raising activities 6. Legal support for GBV survivors 7. Police services for GBV survivors 8. Judicial services for GBV survivors 9. Other **[Please elaborate]** |  |
| 22 | Do you see that any particular groups of women are having more trouble accessing services or are accessing GBV services less during the pandemic? | 1 Yes  0 No  8 Don’t know | 🡪 24  🡪 24 |
| 23 | Which groups of women are having more trouble accessing services or are accessing GBV services less during the pandemic in this country? [Please select all that apply] | 1. Adolescents 2. Women with disabilities 3. Ethnic minorities 4. Migrants, refugees or other displaced people 5. People of diverse sexual orientation, gender identity and expression or sex characteristics 6. Unmarried women 7. Other **[Please elaborate]** |  |
| 24 | Do you think that COVID-19 is impacting the prevalence of GBV and/or intimate partner violence (IPV) in your country? | 1 Yes  0 No  8 Don’t know | 🡪 **27**  🡪 **27** |
| 25 | How has COVID-19 affected IPV/GBV prevalence in this country? | 1. Reports of GBV/IPV have increased 2. Reports of GBV/IPV have reduced 3. Reports of some forms of GBV (such as IPV) have increased, but other forms (such as non-partner violence) have reduced **[Please elaborate]** 4. Other **[Please elaborate]** |  |
| 26 | How did you make these conclusions about the changes in GBV prevalence?  **Please select all that apply** | 1. News/media reports 2. Social media groups 3. Administrative statistics provided by the government 4. Direct reports from women affected 5. Changes in demand for GBV services as indicated by GBV services delivery data 6. Internal organization communications 7. Communications with professional networks 8. Other **[Please elaborate]** |  |
| 27 | Have you seen examples of innovative mechanisms being effectively used to fill some of the gaps in GBV prevention and response in your country? | 1 Yes  0 No  8 Don’t know | 🡪 29  🡪 29 |
| 28 | If yes, please provide an example |  |  |
| 29 | Has the COVID-19 pandemic impacted the provision of sexual and reproductive health (SRH) services in your country? | 1 Yes  0 No  8 Don’t know/ not my area of expertise | 🡪 34  🡪 34 |
| 30 | How has COVID-19 impacted SRH service provision and outreach?  **Please select all that apply** | 1 Full on-site services are available but there has been a reduction in use or demand  2 Full on-site services are available but there has been an increase in use or demand  3 All services have stopped due to lockdown/movement restriction  4. Services have stopped as SRH clinics/services were deemed non-essential by the government  5. Services have stopped/reduced since the beginning of the pandemic due to lockdown/movement restrictions imposed in the country  6. Services were stopped initially due to lockdown/movement restrictions imposed in the beginning of the pandemic, but have since resumed after restrictions were reduced/lifted  7. Services have stopped/reduced as staff have been diverted to emergency response  8.services have stopped/reduced as funding was diverted to emergency response    9. Services have stopped/reduced as there are shortages of personal protective gear and infection management supplies  10. Services have been reduced as use or demand has decreased  11. Services have reduced as staff have additional COVID-19 responsibilities  12.. Services have reduced due to reduced availability of supplies and commodities (for example, due to difficulties with procurement and transport)  13. Service delivery has shifted to remote provision via telemedicine or other innovative strategies  14 Other (please elaborate) |  |
| 31 | What type of SRH services have been limited or stopped since the beginning* of COVID-19?  **Please select all that apply**  **( * by beginning we mean when lockdowns or other restrictive policies were imposed)** | ***Service provision unchanged from beginning (1) Limited service provided from beginning (2) Service stopped completely from beginning (3) Services stopped initially but full services available now (4) Services stopped initially but limited services available now (5) Not applicable/ we do not provide this service (6)***       1. Contraceptive counselling and services 2. Abortion care/ post abortion care 3. Delivery care 4. Antenatal/postnatal care 5. STI screening and treatment 6. HIV testing, care and treatment 7. Reproductive cancer screening 8. Adolescent-friendly or focused SRH services 9. Other SRH Clinical services (Please elaborate) 10. Community outreach and awareness related to SRH 11. Other (Please elaborate) |  |
| 32 | Do you see that any particular groups of women are having more trouble accessing services or are accessing SRH services less during the pandemic? | 1 Yes  0 No  8 Don’t know | 🡪 34  🡪 34 |
| 33 | Which groups of women are having more trouble accessing services or are accessing SRH services less during the pandemic in this country?  **Please select all that apply** | 1. Adolescents 2. Women with disabilities 3. Ethnic minorities 4. Migrants, refugees or other displaced people 5. People of diverse sexual orientation, gender identity and expression or sex characteristics 6. Unmarried women 7. Other **[Please elaborate]** |  |
| 34 | Have you heard that because of Covid-19 women are having difficulty obtaining contraceptives or continuing contraceptive use in your country? | 1 Yes  0 No  8 Don’t know | 🡪 36  🡪 36 |
| 35 | How did you make these conclusions about women’s use of contraception and/or abortion in your country?  **Please select all that apply** | 1. News/media reports 2. Social media groups 3. Administrative statistics provided by the government 4. Direct reports from women affected 5. Changes in demand for our SRH services as indicated by SRH service delivery data 6. Internal organization communications 7. Professional Networks within the field 8. Other (please elaborate) |  |
| 36 | Have you heard that because of Covid-19 women are having difficulty obtaining a safe abortion in your country? | 1 Yes  0 No  8 Don’t know | 🡪 38  🡪 38 |
| 37 | How did you make these conclusions about women’s use of contraception and/or abortion in your country?  **Please select all that apply** | 1. News/media reports 2. Social media groups 3. Administrative statistics provided by the government 4. Direct reports from women affected 5. Changes in demand for our SRH services as indicated by SRH service delivery data 6. Internal organization communications 7. Professional Networks within the field 8. Other (please elaborate) |  |
| 38 | Have you seen innovation and technology being used effectively to cover some of the gaps in SRH service provision in your country? | 1 Yes  0 No  8 Don’t know |  |
| 39 | If yes, please provide an example |  |  |
