# Supplementary material for: COVID-19 and gender-based violence service provision in the United States
Source: PLoS One. 2022 Feb 16;17(2):e0263970. doi: 10.1371/journal.pone.0263970 (PMC8849472; doi:10.1371/journal.pone.0263970)
Supplement: S2 File — (DOCX) [file pone.0263970.s003.docx]

**S2 File. In-depth interview guide**

**Verbal Consent Script– COVID/GBV/SRH Programs and Funding Stakeholder Interview - Nigeria, South Africa, USA**

Participant Duration: 45 minutes Anticipated Number of Subjects: 60

| **Study Title** | COVID-19, gender-based violence, and sexual and reproductive health |
| --- | --- |
| **Investigator** | Terry McGovern, JD, Mailman School of Public Health, Columbia University, USA, Telephone: +1-212-304-5278 |
| **Study Sponsor(s)** | The Ford Foundation |
| **Collaborators** | Neetu John, PhD, Mailman School of Public Health, Columbia University, USA |

**Research Purpose**

We are trying to understand the impact of the COVID-19 pandemic on women, girls, and marginalized groups. We want to learn about the availability and shifts to gender-based violence (GBV) service provision and sexual and reproductive health programs, policies, and funding, in the US, South Africa, Nigeria, Uganda, Kenya, and Colombia. We hope this study will contribute to future responses to GBV and SRH in emergencies and inform policy recommendations regarding how to implement essential GBV and SRH services within emergency preparedness in future pandemics. This study also seeks to identify and promote funding practices that reinforce effective GBV and SRHR responses during emergency responses.

**Information on Research**

**Procedures:** If you agree to participate in this research study, the interview will take approximately 45 minutes. You will be asked some questions relating to your work in gender-based violence and sexual and reproductive health and how it might have changed since the COVID-19 pandemic began. We will record the discussion. The recording will be used for data analysis purposes only. It will not include your name. The recording will be stored on a password protected computer. The recording will be destroyed once the study is completed

**Risks:** The only known risk to you of your involvement in this study is the inconvenience of giving 45 minutes of your time. No information is being collected that could identify you with your responses. We will not write your name anywhere so that your answers cannot be linked to you.

**Benefits:** You will not benefit directly from taking part in this study.

**Confidentiality:** All answers will be kept private. We will not write your name down, and anything you tell us will not be linked to your name. We will do everything we can to keep the data confidential. The results of the study may be published and full quotes from individuals may be used, but quotes will not be linked to named individuals. We will not identify you in anything we write about the study.

**Voluntary Participation**

You are free to join the study or not. You do not have to answer any questions that you do not want to answer. You may stop participating in this study at any time.

**Contact Information:** If you have any questions about the study at any time, you may contact Neetu John at [naj2128@cumc.columbia.edu](mailto:naj2128@cumc.columbia.edu).

***GBV programs and funding context and impact of COVID-19 on GBV sector***

**Stakeholder Interview Guide**

**For NGOs/service providers/program staff/other stakeholders**

**I want to start by asking about your organization and your work:**

- What does your organization do?
  - *[Probes: Who is your target population? Does your organization have a local, statewide, or national scope? What is your approach/strategy? What are your main areas of work? What kinds of services and/or activities do you provide or undertake?]*
- What is your role within the organization?
  - *[Probes: How long have you been working on GBV issues?]*

**Next, I have some questions about how Covid-19 has impacted your work:**

- How has COVID-19 impacted your organization’s work?
- *[Probes: funding? Operations? Staff? Relationships with other stakeholders?]*
- What has been the impact on the services and/or activities you provide or undertake?
- *[Probes: disruption/reduction of services/activities? Change in modality?]*
- What has been the impact on your role?
- *[Probe: Does your typical day look different now from the pre-COVID days?]*
- What are some key factors that have allowed (or prevented) you and your organization to continue to function (or not) given the pandemic?
  - *[Probes: Restrictive policies such as lockdowns and movement restriction; other policies such as deeming GBV services non-essential; funding loss/reduction; reduced or increased demand and use of services; shortage of essential supplies, shortage of personal protective gear; shortage of staff/space due to divergence to emergency response; innovative technologies and tele-health; shifts in public or officials' opinion toward the organization or its areas of work]*
- Have you noticed any changes in demand and use for services/products? Why or why not?
  - *[ Probes: Have you seen a surge in IPV and other forms of GBV? ]*
- Who would you say are the overlooked groups when it comes to GBV service provision? Are certain categories of women and girls finding it even harder to access services and prevention activities? Why or why not?
  - *[Probe: Adolescents; Women with disabilities; Ethnic minorities; Migrants, refugees or other displaced people; People of diverse sexual orientation, gender identity and expression or sex characteristics; Unmarried women]*
- What has been the impact on GBV funding during the pandemic?
- *[Probes: Is funding being reallocated away from GBV services and prevention (or other essential services women and girls need) due to the pandemic? Where is it being diverted to (types of activities and types of recipients, e.g. is it mostly going to government agencies? Hospitals?)? What types of funding are most impacted?*
- Overall, are you satisfied with the kinds/level of GBV services and prevention activities being made available to women and girls? Why or why not?

**Now I have several questions about policies during the response to the Covid-19 pandemic, as well as the larger policy landscape.**

- How has your organization juggled discrepancies between local, state, and federal restrictions/policies during the response to the Covid-19 pandemic?
- How have state and local governments responded to GBV needs of women and girls during the pandemic?
  - *[Probes: Have GBV services or prevention activities been deemed non-essential or have state and local governments been pro-active and put in place policies and guidelines to ensure women and girls continue to access GBV services and prevention activities? Why or why not?*
- There have been rapid changes to the policy landscape over the last few years. How do you think the pandemic and response have intersected with the pre-pandemic policy landscape?
  - [Probes: Title X changes, immigration policy changes, protections for LGBTQ+, etc.]
- The Black Lives Matter protests earlier this summer incited what many are calling a “racial reckoning” in the United States, calling attention not only to racial disparities in policing but also with regard to health access and outcomes. Has the BLM movement impacted your work at all? If so, in what way?

**Now I want to ask about funding for GBV organizations like yours:**

- Who are your main donors?
  - *[Probes: what type of donor (i.e. private foundations, government, bilateral donors)*
- What kinds of funding do you receive? What is their typical duration and mechanism? and how does their mechanism and duration impact your work?
  - *[Probes: kind of funding: general support, grants, contracts, subcontractor, upfront payment, or reimbursement basis]*
- Do you collect monitoring and evaluation indicators as part of donor requirements to track progress? Do you face challenges because of these requirements?
  - *[ Probes: Is there a specific type of donor who makes these requirements? Do you find this collected information useful? Besides donor reporting, how do you use the information? Is there a contradiction between these donor requirements and grass-root realities of managing a program?]*
- Overall, can you reflect on some key bottlenecks in terms of donor requirements and funding streams that make it harder for you to reach the women and girls you serve?
  - *[ Probe: Is there GBV work that you’d want to do but can’t, and why?; What would make it easier for you to do this work? ; Any recommendations specific to donor strategies or practices?]*
- In your opinion, do donor priorities reflect the needs on the ground? What is working and what is not working?
  - *[ Probe: Do you have key recommendation on what funders/donors could change that would make your work more impactful?]*

**I would like to wrap up with a couple of broad questions about GBV in the United States. We would love to hear your perspective as a practitioner with expertise in the field, beyond the work of your specific organization.**

- What is your assessment of the state of GBV prevention and response in the United States?
  - *[Probe: Key successes? Key challenges? Vulnerable Populations -- Who is missing - Migrant women, Women with disabilities, LGBTQ, Adolescents, and young women? What needs to change?]*
- How has COVID-19 impacted GBV service provision and prevention/awareness raising work in the United States?
  - *[General Probe: What services and prevention activities continue to be available? What has stopped? If there are bottlenecks in the prevention and response system, what are they?]*
  - *[services Probe: Are clinical, counseling and case management services available? Are shelters available and safe? Are police and judicial systems responsive to complaints of GBV (e.g. are police registering complaints and enforcing protection orders; are courts hearing cases and issuing protection orders, child support/alimony/divorce orders)? How has community-based prevention and awareness-raising activities been impacted?]*
  - *Have you seen examples of innovative mechanisms being effectively used to ensure continuity of services?*
- Why has there been such an impact?
- *[Probe: Could something have been done to change the outcome?]*

**Do you have any questions for me before we wrap up?**
