# Supplementary material for: COVID-19 and gender-based violence service provision in the United States
Source: PLoS One. 2022 Feb 16;17(2):e0263970. doi: 10.1371/journal.pone.0263970 (PMC8849472; doi:10.1371/journal.pone.0263970)
Supplement: S3 File — (PDF) [file pone.0263970.s004.pdf]

Request for Permission to Publish Content under CC-BY License

Dear Rights Holder or Representative,

I have submitted a paper for publication in a PLOS journal, and wish to include the content listed below in the paper. I'm hereby requesting your (or your company's or institution's) permission to include the content in my paper. Please note that all PLOS journals are published under a Creative Commons Attribution License (CC BY), which allows for unrestricted use and distribution, even commercial, as long as attribution is given to the creator or rights holder of the content. See https://creativecommons.org/licenses/by/4.0/.

To grant me permission to use the content in my PLOS paper, please fill in the information below and then scan the completed form and send it to me at my email address.

Thank you.

My name:

Rachel Sapire

My email address:

rjs2175@columbia.edu

Description of the content which I'm seeking permission to use (citation and/or title, and pasted screen shot, if applicable):

Fig 1. U.S. states that exempted some or all GBV survivors from COVID-19 emergency measures.

Link to the Content:

n/a (attached to email)

* * *

On behalf of myself or the rights holder, I hereby grant the permission sought herein.

Signature of Party Granting Permission:

Date:

4 Jan 2022

Printed Name and Title:

Minas Giannekas, owner and developer of MapChart (https://mapchart.net)

Rachel Sapire <rjs2175@caa.columbia.edu>

Request for Permission to Publish Map on PLOS ONE

2 messages

Rachel Sapire <rjs2175@caa.columbia.edu> Mon, Jan 3, 2022 at 4:10 PM
To: contact@mapchart.net

Hello,

I hope you are well.

We utilized your site to create the attached map (Fig1) for a paper submitted to PLOS ONE for publication. I understand that you give permission on your site to publish maps, however, the CC-BY-SA-4.0 license that mapchart.net uses is not compatible with the license PLOS uses. (PLOS publishes all content under the Creative Commons Attribution (CC BY) 4.0 license, which means that they will be freely available online, and any third party is permitted to access, download, copy, distribute, and use these materials in any way, even commercially, with proper attribution.)

As such, I am writing to formally request permission for the open-access journal PLOS ONE to publish the attached map of the United States, highlighting states by exceptions for GBV survivor travel under state COVID-19 restrictions (Fig1), under the Creative Commons Attribution License (CCAL) CC BY 4.0 (http://creativecommons.org/licenses/by/4.0/). Please be aware that this license allows unrestricted use and distribution, even commercially, by third parties.

Please reply and provide explicit written permission to publish the map under a CC BY license *and* return the attached form.

We really appreciate your help and assistance with this process.

Warmly,

Rachel Sapire
rjs2175@columbia.edu

2 attachments

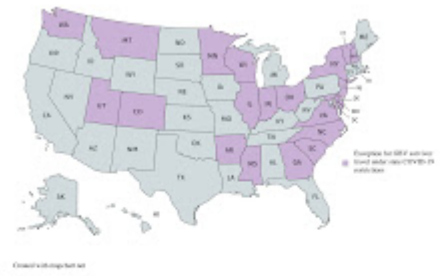

Fig1.tif
601K

content-permission-form_MapChart.pdf
277K

MapChart <contact@mapchart.net> Tue, Jan 4, 2022 at 2:17 AM
Reply-To: contact@mapchart.net
To: Rachel Sapire <rjs2175@caa.columbia.edu>

Hi Rachel,

Thank you for contacting me. I am so glad that my website was helpful for your project. Please find attached the signed permission form.

If you need anything else, please let me know. Good luck with your publication!

[Quoted text hidden]

content-permission-form_MapChart_signed.pdf
348K
